# Calculation of organ doses from breast cancer radiotherapy: a Monte Carlo study

**DOI:** 10.1120/jacmp.v14i1.4029

**Published:** 2013-01-07

**Authors:** T. Berris, M. Mazonakis, J. Stratakis, A. Tzedakis, A. Fasoulaki, J. Damilakis

**Affiliations:** ^1^ Department of Medical Physics Faculty of Medicine, University of Crete Heraklion, Crete Greece; ^2^ Department of Medical Physics University Hospital of Heraklion Heraklion, Crete Greece; ^3^ Department of Radiotherapy and Oncology University Hospital of Heraklion Heraklion, Crete Greece

**Keywords:** breast cancer, radiotherapy, Monte Carlo simulation, organ doses

## Abstract

The current study aimed to: a) utilize Monte Carlo simulation methods for the assessment of radiation doses imparted to all organs at risk to develop secondary radiation induced cancer, for patients undergoing radiotherapy for breast cancer; and b) evaluate the effect of breast size on dose to organs outside the irradiation field. A simulated linear accelerator model was generated. The in‐field accuracy of the simulated photon beam properties was verified against percentage depth dose (PDD) and dose profile measurements on an actual water phantom. Off‐axis dose calculations were verified with thermoluminescent dosimetry (TLD) measurements on a humanoid physical phantom. An anthropomorphic mathematical phantom was used to simulate breast cancer radiotherapy with medial and lateral fields. The effect of breast size on the calculated organ dose was investigated. Local differences between measured and calculated PDDs and dose profiles did not exceed 2% for the points at depths beyond the depth of maximum dose and the plateau region of the profile, respectively. For the penumbral regions of the dose profiles, the distance to agreement (DTA) did not exceed 2 mm. The mean difference between calculated out‐of‐field doses and TLD measurements was 11.4%±5.9%. The calculated doses to peripheral organs ranged from 2.32 cGy up to 161.41 cGy depending on breast size and thus the field dimensions applied, as well as the proximity of the organs to the primary beam. An increase to the therapeutic field area by 50% to account for the large breast led to a mean organ dose elevation by up to 85.2% for lateral exposure. The contralateral breast dose ranged between 1.4% and 1.6% of the prescribed dose to the tumor. Breast size affects dose deposition substantially.

PACS numbers: 87.10.rt, 87.56.bd, 87.53.Bn, 87.55.K‐, 87.55.ne, 87.56.jf, 87.56.J‐

## I. INTRODUCTION

Breast tumors are the most common form of cancer in female patients of European countries, accounting for 30.9% of all incident cases.^(^ ^1^ ^)^ The early cancer diagnosis with screening mammography and the recent advances in adjuvant therapy have reduced the mortality from breast malignancies.^(^ ^2^ ^,^ ^3^ ^)^ The five‐year survival rate of women suffering from breast carcinomas has reached to 79% in Europe.^(^ ^4^ ^)^ Radiation therapy has a major role in the adjuvant management of breast carcinoma.^(^ ^5^ ^)^ However, radiotherapy inevitably exposes to ionizing radiation the organs/tissues that are entirely or partially excluded from the treatment volume. Berrington et al.^(^ ^6^ ^)^ found a high relative risk for lung and esophageal cancer induction due to the high radiation dose of more than 1 Gy received by these organs. Stovall et al.^(^ ^7^ ^)^ showed that women below 40 years of age have an elevated risk for developing contralateral breast cancer when the healthy breast dose exceeds 1 Gy. Huang and Mackillop^(^ ^8^ ^)^ reported a high risk for radiotherapy‐induced soft tissue sarcomas in the vicinity of the treated anatomic region. Therapeutic irradiation might be associated with a relatively increased risk for carcinogenesis in distant sites from the breast, such as salivary glands and ovaries, and it also has a significant effect on gynecological cancer incidence.^(^ ^8^ ^,^ ^9^ ^)^

Scarce information exists in the literature about organ doses due to breast cancer radiotherapy. Previous studies dealing with healthy organ dose calculation from breast radiotherapy were performed using treatment planning systems and, therefore, their results were restricted to anatomic sites located very close to the treatment field.^(^ ^10^ ^–^ ^12^ ^)^ Both *in vivo* and phantom measurements with thermoluminescent dosimeters (TLD) have also been employed for organ dose determination from breast cancer treatment.^(^ ^6^ ^,^ ^7^ ^,^ ^13^ ^,^ ^14^ ^)^ *In vivo* experiments are limited to surface dose estimation. Phantom measurements require the placement of numerous TLD chips in a tissue‐equivalent phantom to detect either the dose variation across each organ volume or the steep dose gradients observed in organs that were partly included in the primary beam. Thermoluminescent dosimetry may be considered as a time‐consuming and labor‐intensive procedure, often prone to high uncertainties. Furthermore, to the best of our knowledge, no study has yet dealt with the assessment of radiation dose to all organs at risk (OAR) to develop radiation induced cancer as recently defined by the International Commission on Radiological protection (ICRP).^(^ ^15^ ^)^ The computerized Monte Carlo (MC) technique uses mathematical phantoms with realistic anatomic proportions, and it can provide a mean organ dose value by taking into account the radiation exposure inside the entire organ volume. Depending on the phantom employed, dose to all OARs for radiation induced cancer is feasible. Such dose estimations are useful from a radiation protection perspective.

The current study was conducted to: a) simulate breast radiotherapy with a megavoltage beam using the Monte Carlo N‐particle (MCNP) transport code in order to calculate the radiation dose to all OARs of patients undergoing radiotherapy for breast cancer, and b) evaluate the effect of breast size, and subsequently irradiation field size, on peripheral organ doses.

## II. MATERIALS AND METHODS

### A. Linear accelerator modeling

**Figure 1:**
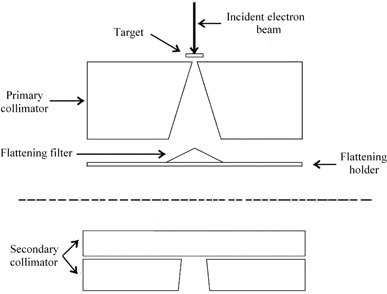
Descriptive representation of the linear accelerator (linac) simulation. The dashed line divides the geometry conceptually and specifies the linac parts included in each simulation step.

A simulated model of a 6 MV medical linear accelerator (linac) (Philips SL 75/5, Philips/Elekta, The Netherlands) was generated using the MCNP radiation transport code.^(^ ^16^ ^)^ The model incorporated the basic beam modifying components, namely the heavy metal target, primary collimator, flattening filter, flattening filter holder, and secondary collimator. The exact geometry and materials specification of the linac head was derived by detailed blueprints provided exclusively by the manufacturer of the radiotherapeutic unit. To save simulation time, the implementation of the MC model was performed in two steps.^(^ ^17^ ^)^ The linac parts included in the simulation are depicted in Fig. 1.

In the first step of the linac simulation, a 6 MeV electron beam impinged perpendicularly on the heavy metal target. The energy spread and the radial intensity distribution of the electron beam were supposed to be 1 MeV and 1 mm full width at half maximum (FWHM), respectively.^(^ ^18^ ^)^ The produced bremsstrahlung photons passed through the primary collimator and their forward peaked intensity distribution was compensated by the flattening filter. A circular tallying surface was defined just under the flattening filter holder. The surface was divided into 13 annular rings using the tally segmentation feature of the MCNP code. The width of each ring was 0.2 cm. The outer annular ring extended to the boundaries of the photon field defined by the extension of the conical surface, which incorporated the inner surface of the primary collimator. An F2 type tally was assigned to every annular ring. The flux of the photons crossing each ring was calculated and registered separately for 250 keV energy bins that spanned an energy range from 0 to 8 MeV. The energy cutoff feature of the MCNP code was used as a variance reduction technique. The cutoff energies were set to 10 and 500 keV for photons and electrons, respectively.^(^ ^17^ ^)^ The number of source electrons needed for the detectors to produce statistically meaningful results with a relative error below 1% was $10^{9}$. Many of those electrons performed more than $10^{4}$ individual interactions before any kind of a cutoff occurred. An equal number of calculations was performed by the code in order to track their histories.

In the second step of the simulation, the photon spectra calculated by each annular ring were re‐emitted by a point source at the azimuthal angular intervals corresponding to each ring. The MCNP code provides an automatically uniform polar sampling for photon emission directions and, therefore, no polar photon emission parameters were specified. The secondary collimator was also included in the second part of the simulation. The distance between the blocks of the simulated secondary collimator and the point source was equal to the distance between the actual secondary collimator blocks and the upper surface of the target where the electron beam strikes. The conical beam emitted from the point source could be shaped into rectangular fields of any dimension with the aid of the simulated secondary collimator.

### B. Mathematical phantom

The BodyBuilder commercial software (version 1.30, White Rock Science, Los Alamos, NM) was used for the generation of an androgynous mathematical phantom with a height of 1.79 m and weight of 73.54 kg.^(^ ^19^ ^)^ The mathematical phantom included 21 of the 29 “main” and “remainder” OARs recently defined by the ICRP.^(^ ^15^ ^)^ The eight missing organs were the following: prostate gland, salivary glands, lymph nodes, red bone marrow (RBM), bone surface (BS), extra‐thoracic tissue, muscle, and oral mucosa.

The radiation exposure of salivary glands was estimated by appropriate tally cells added to the three‐dimensional geometry in accordance with the Van Ripper et al. report.^(^ ^20^ ^)^ The radiation dose imparted to the soft tissue of the neck of the mathematical phantom was taken as the extrathoracic tissue dose. Muscle exposure was determined by the mass weighted mean doses imparted to all phantom's tissues, excluding those of specifically defined organs.^(^ ^21^ ^)^ The mean radiation dose to the oral mucosa was assumed to be equal to the dose received by the sublingual salivary glands.

The cells of the mathematical phantom corresponding to the skeleton consisted of solid bone tissue. There were no soft tissue structures to represent the RBM tissue. The mean dose imparted to the RBM, $D_{RBM}$ was approximated by the equation:

(1) $${\bar{D}}_{RBM}={\bar{D}}_{Bone}\cdot \frac{(\frac{\mu_{en}}{\rho })RBM}{(\frac{\mu_{en}}{\rho })Bone}$$

where ${\bar{D}}_{Bone}$ is the mean dose to the solid osseous tissue, and the notation $\frac{\mu_{en}}{\rho }$ corresponds to the mass‐energy absorption coefficient of a material.^(^ ^22^ ^)^ RBM tissue was considered to match the composition of soft tissue. The selected values of the mass‐energy absorption coefficients for bone and soft tissue corresponded to the mean energy of the photon beam. The mean energy of the photon spectrum was calculated in the previously described first step of the linac simulation. The fractions of active RBM residing in various osseous structures needed for the calculation of the total organ dose were obtained by a previously published report.^(^ ^21^ ^)^

The mathematical phantom had no specifically defined cells to represent the BS (i.e., the approximately 50 μm thick layer of trabecular endosteum of each bone^(^ ^23^ ^)^. The BS dose was considered to be equal to the dose received by the solid osseous tissue. This approach should give a relatively accurate dose estimate in the photon energy range above about 100 keV.^(^ ^24^ ^)^ BS was considered to be uniformly distributed throughout the skeleton.

The lymph node distribution in the mathematical phantom was approximated with the aid of a three‐dimensional atlas of lymph node topography.^(^ ^25^ ^)^ The dose to the lymph nodes was estimated either by the mass‐weighted radiation doses received by organs or by specifically defined tally cells distributed in the lymphoid regions of the simulated human body (Table 1).

**Table 1:** Lymphatic distribution in the mathematical phantom and regions of dose calculation to lymph nodes.

*Lymph Node Cluster*	*% of the Total Lymph Node Volume*	*Region of Dose Calculation*
Head & neck	1%	Parotid salivary glands, thyroid^a^
Head & neck	24.1%	Neck soft tissue^a^
Chest	8.8%	Heart^a^
Axilla	6%	Left and right axilla^b^
Abdomen	2.5%	Stomach^a^
Abdomen	41.1%	Small intestine^a^
Pelvis	13.5%	Upper pelvis^b^
Pelvis	3%	Upper leg muscle^b^

a The radiation dose to lymph nodes was approximated by the mass‐weighted organ doses.

b Tally cells were added for lymph node dose calculation.

The arms of the mathematical phantom used in this study were incorporated in the trunk. To represent the real irradiation of the left breast, a left arm was designed and positioned over the mathematical phantom's head. The osseous tissue of the left arm bone which was originally inside the trunk was substituted with soft tissue.

### C. Verification of the simulated beam model

#### C.1 Comparison of Monte Carlo results with ionization chamber measurements

The setup of the second part of the simulation was utilized for the calculation of percent depth dose (PDD) of the MC beam model. Depth dose curves were calculated for square $10\times 10\;{\text{cm}}^{2}$ and $20\times 20\;{\text{cm}}^{2}$ radiation fields on a simulated $50\times 50\;50\times 50\;{\text{cm}}^{3}$ water phantom. The SSD was set to 100 cm and *F8 type tally cells were embedded in regular distances inside the water phantom volume to score the energy deposition at various depths. The tally cells were cylindrical and coaxial with the simulated therapeutic beam. Their height was equal to 0.2 cm. A radius of 1 cm was chosen to increase tally cell surfaces that were perpendicular to the beam's axis and to subsequently improve the simulation efficiency. The number of source photon histories needed to achieve a relative error less than 1% for all detectors was $2.4\times 10^{8}$.

For the same field sizes, beam profiles were calculated using an array of cylindrical F6 type tallies embedded in the water volume. F6 tallies calculate collision kerma, which is a quantity proportional to the dose at depths equal to the depth of maximum dose ($d_{\max}$) and beyond. The axes of the tally cells were laid on the middle plane of the water phantom and they were parallel to the beam's axis. The cylinders had a radius of 0.5 cm and they were 1 cm long. The array could be moved along the direction of the beam's axis, and dose profiles were calculated at $d_{\max}$ and at a depth of 10 cm ($d_{10}$). The $d_{\max}$ was determined based on the previously described PDD calculations. A detector error below 1% was obtained by using $2\times 10^{8}$ source photon histories. The calculated PDDs and dose profiles were directly compared with corresponding curves generated on a physical water phantom (RFA‐300, Scanditronix Wellhofer, Uppsala, Sweden) with dimensions $49.5\times 49.5\times 49.5\;{\text{cm}}^{3}$. Ionization measurements were made with a 0.13 cc calibrated chamber (CC13‐S, Scanditronix Wellhofer). The dose evaluation to the penumbral, high‐dose gradient parts of the beam profiles was performed with the distance to agreement (DTA) method.^(^ ^26^ ^)^

#### C.2 Comparison of Monte Carlo results with TLD measurements

A RANDO humanoid phantom (Alderson Research Labs, Stanford, CA) and thermoluminescence dosimetry were employed to measure out‐of‐field radiation doses. The phantom has been constructed by tissue‐equivalent material and it represents a human body trunk from the upper third of the thighs to the vertex of an average individual with a height of 173 cm and a weight of 73.5 kg. A left breast made of wax was placed on the RANDO phantom.^(^ ^27^ ^)^ The dimensions of the breast were similar with those of the tissue in the mathematical phantom. The physical phantom was irradiated with the same setup used in MC simulations. The tangential field size used was $10\times 16\;{\text{cm}}^{2}$.

Calcium fluoride thermoluminescent dosimeters (TLD‐200, Harshaw, Solon, OH), suitable for scattered dose measurements, were positioned in the RANDO phantom. The calibration of the crystals was made on the same linear accelerator with that used for direct measurements and modeled by the MCNP code. The procedure of the TLD calibration and reading has been previously described.^(^ ^28^ ^)^ The standard deviation of the sensitivity factors was less than 4% for the TLD batch used in this study. The crystals were positioned at distances of 15 cm, 20 cm, 25 cm, 30 cm, 35 cm, and 40 cm from the field isocenter. The depth from the anterior phantom's surface varied from 9 to 11 cm. The TLD measurements were compared with out‐of‐field dose calculations. Six spherical MCNP F6 tallies with a radius of 1 cm were positioned inside the mathematical phantom at the same distances and depths with those referred for TLD crystals. The simulations performed needed $2\times 10^{8}$ histories to achieve an error less than 7% for all tallies.

### D. Breast radiation therapy simulation

The second part of the linac beam simulation in conjunction with the mathematical phantom was used to determine organ doses attributable to tangential irradiation of the left breast consisting of a pair of medial and lateral fields. The effect of the breast size on the out‐of‐field organ was investigated in our study. Dose calculations were initially carried out using the standard field dimensions of $10\times 16\;{\text{cm}}^{2}$. Moreover, organ doses were determined using an increased field size of $12\times 20\;{\text{cm}}^{2}$ coupled to a large‐sized breast to evaluate the effect of scattering on a larger breast to the peripheral organ doses. To achieve this simulation, the original breast size of the phantom, which represents a small breast of $168.50\;{\text{cm}}^{3}$, was changed to $1354.4\;{\text{cm}}^{3}$ to represent very closely the average large breast size.^(^ ^29^ ^)^ Similar ranges of breast sizes, as well as irradiation field sizes, have been documented elsewhere in the literature.^(^ ^30^ ^,^ ^31^ ^)^ For both breast sizes, the tumor site was located at the center of the left breast tissue and the source‐to‐skin distance (SSD) was set to 100 cm. The gantry angles for the medial and the lateral treatment field corresponding to the small breast size were 286° and 115°, respectively. Due to the different anatomy of the phantom with large breast size, the respective medial and lateral field angles were 301° and 128°. In all cases the opposing posterior field borders were aligned. The contralateral breast was very close, but not exposed, to the primary beam. This setup ensured the detection of the maximum scattered dose to the contralateral breast. The treatment setup was defined by a radiotherapist experienced in the management of breast carcinoma. Organ doses were calculated for equally weighted field irradiations giving 50.4 Gy to the tumor site.

Doses to OARs were calculated by assigning an F6 type tally to each geometrical cell representing an organ or a part of it. The F6 tally calculates the average collision kerma at every geometrical cell. In this study, the approach of Gu et al.^(^ ^32^ ^)^ was followed and collision kerma was considered equal to absorbed dose for the relatively low energy of 6 MV photons. The dose to each organ was calculated as a weighted average of the doses received by the various fractions of the organ throughout the phantom's body. Organs or organ parts exposed by the primary beam were excluded from dose calculation.^(^ ^33^ ^)^ The dose to the RBM and the BS was calculated excluding the dose received by the ribs. Lung exposure was approximated by the dose to the right lung, since the left lung was partially inside the primary beam. The dose to the trunk and left breast skin was not taken into account for the total skin dose calculation. The radiation dose to tissues between the ribs and skin was also excluded from the determination of muscle exposure. The breast dose corresponded to the contralateral breast, which was outside the primary photon beam. The relative error of all dose calculations was kept below 7% even for cells far outside the primary beam. To keep the error that low, the number of source photon histories was $2\times 10^{8}$ for each projection.

## III. RESULTS

### A. Linear accelerator modeling

**Figure 2:**
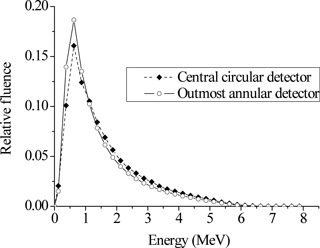
Photon spectra calculated by the central circular and the outmost annular detector. The photon spectrum was “softer” towards the edges of the field.

**Figure 3:**
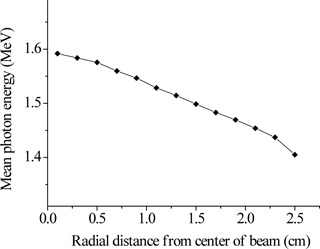
The mean energy of the photon spectra calculated by each one of the annular detectors just below the flattening filter holder.

The photon spectrum passing through the flattening filter holder was found to be “softer” as the distance from the central beam axis increased. Figure 2 shows the spectrum obtained by the central circular detector and the outer annular detector to illustrate the variation of the photon spectrum as a function of the distance from the beam's axis. The outmost annular detector received relatively more low‐energy photons than the central detector. In contrast, the central detector collected more high‐energy photons than the outer ring. Figure 3 shows the mean photon energy observed at various distances from the beam's axis at the detectors level exemplifying the radial softening of the spectrum. The mean energy of the spectrum calculated by the outmost detector was found to be 11.7% lower than that calculated by the central circular tally. The total photon fluence just below the flattening filter holder was 3.5 photons for every 100 source electrons. These results are in agreement with previously published data.^(^ ^17^ ^)^

### B. Verification of the simulated beam model

**Figure 4:**
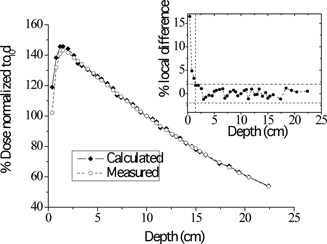
Superimposed measured and calculated percentage depth dose (PDD) curves for the $20\times 20\;{\text{cm}}^{2}$ photon field. The inset shows the local differences of the calculated values from the corresponding measured PDD values. The horizontal dashed lines mark the ±2% limit of acceptance. The vertical dashed line marks the depth of maximum dose.

**Figure 5:**
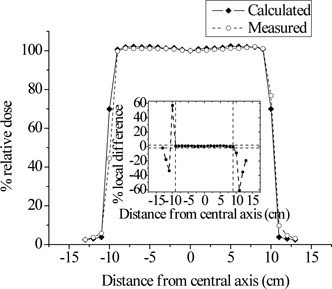
Superimposed measured and calculated lateral dose profiles for the $20\times 20\;{\text{cm}}^{2}$ photon field at depth of maximum dose ($d_{\max}$). The inset shows the local differences of the calculated values from the corresponding measured dose profile values. The horizontal dashed lines mark the ±2% limit of acceptance. The vertical dashed lines limit the plateau region of the profiles.

**Figure 6:**
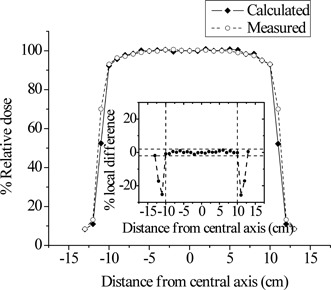
Superimposed measured and calculated lateral dose profiles for the $20\times 20\;{\text{cm}}^{2}$ photon field at 10 cm depth ($d_{10}$). The inset shows the local differences of the calculated values from the corresponding measured dose profile values. The horizontal dashed lines mark the ±2% limit of acceptance. The vertical dashed lines limit the plateau region of the profiles.

Figure 4 shows a comparison of the calculated and measured PDD curve for the $20\times 20\;{\text{cm}}^{2}$ field. The doses were normalized to $d_{10}$. Local differences at depths beyond $d_{\max}$ were less than 2%. Similar results were also obtained for the smaller field size of $10\times 10\;{\text{cm}}^{2}$. Figures 5 and 6 present superimposed measured and calculated dose profiles for the $20\times 20\;{\text{cm}}^{2}$ radiation field at $d_{\max}$ and $d_{10}$, respectively. In the plateau region of the profiles, the local differences did not exceed the value of 2%. For the collimated field of $10\times 10\;{\text{cm}}^{2}$, the maximum local difference between measured and calculated profiles was also less than 2% in the plateau region. In the penumbra region of all profiles considered, spatial differences (DTA) up to 2 mm were observed between calculations and measurements. The above discrepancy should be considered as acceptable.^(^ ^34^ ^)^ Regarding the out‐of‐field point doses, no systematic differences were observed between the MCNP calculations and TLD measurements. The mean difference was equal to 11.4%±5.9%.

### C. Breast radiation therapy simulation

The mean radiation doses to all OARs associated with radiotherapy for breast cancer with tangential lateral and medial fields are summarized in Table 2. These tabular data show the organ dose variation with the breast size and the field size employed. The mean dose increase was found to be equal to 67.4% and 85.2% for the medial and lateral field irradiations, respectively. For a treatment course delivering 50.4 Gy to the tumor site with a field size of $10\times 16\;{\text{cm}}^{2}$, organ dose varied from 2.32 cGy in urinary bladder to 161.41 cGy in heart. Dose variation with the increased field dimensions of $12\times 20\;{\text{cm}}^{2}$ ranged from 4.06 cGy in urinary bladder to 141.59 cGy to the thymus. The heart and thymus were the remainder organs receiving the largest amounts of radiation. The highest exposure to a main organ was observed for the contralateral breast with an absorbed dose of 69.57 cGy to 83.10 cGy, depending upon the breast size and the matching field size employed. The absorbed dose to the contralateral breast was 1.4% and 1.6% of the prescribed dose to the tumor for the small and large breast radiotherapy simulation, respectively.

**Table 2:** Organ doses due to radiotherapy for breast cancer using two different field sizes.

	*Organ Dose (cGy)*			
	*Lateral Field*		*Medial Field*	
	$10\times 16\;cm^{2}$	$12\times 20\;cm^{2}$	$10\times 16\;cm^{2}$	$12\times 20\;cm^{2}$
*Main organs*				
Female breast	37.05	50.18	32.53	32.92
Stomach	16.63	41.89	14.77	36.25
Esophagus	17.98	33.00	13.94	20.49
Thyroid	9.97	21.58	11.28	16.93
Liver	9.22	16.20	7.55	10.18
Red bone marrow	7.28	13.20	5.93	9.61
Lung	4.90	7.06	4.82	5.04
Bone surface	4.22	7.48	3.76	6.08
Salivary glands	4.35	7.65	3.87	5.98
Colon	2.39	4.39	2.43	4.01
Skin	1.96	6.73	2.05	7.95
Brain	2.06	3.38	1.64	2.63
Ovaries	1.58	3.14	1.51	2.53
Urinary bladder	1.04	1.73	1.28	2.33
*Remainder Organs*				
Heart	83.49	86.18	77.93	44.84
Thymus	84.24	102.88	41.67	38.71
Pancreas	12.24	25.39	10.04	19.83
Spleen	11.27	24.23	10.48	25.26
Lymphatic nodes	10.96	14.83	10.42	10.60
Adrenals	8.83	17.46	6.69	11.63
Gall bladder	5.82	11.04	5.90	8.52
Extra‐thoracic tissue	5.70	10.82	5.12	8.00
Kidneys	4.87	9.27	3.65	6.55
Oral mucosa	3.63	7.12	3.59	5.61
Small intestine	2.66	4.90	2.67	4.41
Uterus	1.56	2.71	1.59	2.66
Muscle	1.24	2.44	1.27	3.34

## IV. DISCUSSION

In this study, a MC method was employed to produce a model of a 6 MV medical linac photon beam. The two‐step implementation of the model provided a photon beam that could be used for any radiotherapy simulation without the need to resimulate the whole linac geometry. The measured and calculated PDD values were in a very good agreement at depths beyond $d_{\max}$, with local differences not exceeding 2%. Relatively large discrepancies up to 16% were observed at depths shallower than $d_{\max}$. Similar discrepancies reaching up to 15%, attributed to perturbations caused by the measurement chamber and inaccurate simulation of the treatment head, have been previously observed.^(^ ^18^ ^,^ ^35^ ^–^ ^38^ ^)^ The calculated dose values agreed very well with the measured ones in the plateau regions of the profiles with differences of less than 2%. The discrepancies observed at the abrupt dose fall‐off regions of the profiles might be due to the poor sampling achieved by the relatively large tally detector sizes used in MC simulations. It should be noticed that the differences between measurements and calculations in the profile regions with high‐dose gradients were found to be acceptable based on the 2 mm criterion that was also used by Bednarz and Xu.^(^ ^34^ ^)^ The moderate agreement between the measured and calculated out‐of‐field doses should be attributed to the uncertainty of TLD dosimetry, to error in MC calculations, and to differences in size and composition of the RANDO phantom with the mathematical humanoid phantom.

The absorbed dose to OARs from left breast radiotherapy was calculated in this study. Treatment of the right breast may lead to different dose values, especially for organs not located in the patient's midline. For instance, heart dose from right‐breast irradiation may be lower than that found in this report. Tangential field irradiation is commonly used during breast cancer management.^(^ ^5^ ^)^ As expected, higher doses were observed to organs located close to the irradiated volume. Deep seated organs close to the beam edge, such as heart, thymus, pancreas, spleen, esophagus, stomach, brain, adrenals, kidneys, and liver, received a higher dose from lateral field irradiation than that associated with a medial portal. This might be attributed to the beam broadening inside the trunk and to the subsequent increase of scattered radiation arising within the phantom's body. For organs positioned close to anterior phantom's surface, such as the urinary bladder, the radiation dose from medial fields was higher than that calculated from lateral portals. The above might be due to the contribution of collimator scatter and linac head leakage to the total organ dose that comes from internal scattering within the phantom. This assumption is reinforced by the fact that deep‐seated organs located far away from the treatment field, such as the uterus, received almost the same radiation dose from both projections. Lower energy and thus less penetrating collimator scattered and leakage radiation could not reach them and add to their total dose. Kase et al.^(^ ^39^ ^)^ have shown that head leakage may constitute an important contribution to the total dose of organs far away from the beam's boundaries. Although the dose imparted by leakage radiation is very small, it becomes the dominant contribution for distances beyond 60 cm from the central axis of the beam.

Dose to peripheral organs was generally higher for the large breast size due to increased field size, as well as increased scattering arising from a larger portion of the body in the primary beam. The lower dose that was registered for the heart in the large breast radiotherapy simulation was due to the increase of the distance between the heart and the primary beam. The distance was changed when the dimensions of the field and angles of irradiation were changed to incorporate the whole large left breast while avoiding the contralateral breast.

Dosimetric calculations were carried out for radiotherapy of a standard breast size corresponding to an average patient. Furthermore, an increased breast size was used in order to obtain the most conservative organ dose values. To the best of our knowledge, there are few studies in the literature providing results directly comparable to the results presented herein. Van der Giessen^(^ ^40^ ^)^ measured peripheral doses in a water phantom. Simulations of breast radiotherapy were carried out by adding tissue‐equivalent breast phantoms representing three sizes of breasts. The breast representing the medium breast size in that study was close in dimensions with the large breast in the present study. In this study, 0.1% of the prescribed dose was imparted to the uterus. The center of the uterus is at a distance of about 38 cm from the middle plane of the beam. For the same distance, Van der Giessen's study determined a dose equal to about 0.085%. Kelly et al.^(^ ^41^ ^)^ measured radiation doses to the contralateral breast from different irradiation techniques by placing TLD chips inside the breast of a RANDO phantom. Their results for TLD measurements at the central portion of the breast were comparable with the contralateral breast doses of 1.6% presented here.

Doses to contralateral breast from breast cancer radiotherapy are higher than those from computed tomography and other imaging modalities. According to Parker et al.,^(^ ^42^ ^)^ diagnostic chest CT may impart doses ranging from 2 cGy to 5 cGy to the female breast. Hurwitz et al.^(^ ^43^ ^)^ have reported breast doses up to 6 cGy from body 16 multidetector CT protocols. This is of course a large dose in comparison with the typical 0.2 cGy dose arising from mammography,^(^ ^42^ ^)^ but it is also more than an order of magnitude lower than doses to the contralateral breast from breast radiotherapy, as discussed in this work.

The current available risk factors for radiation‐induced cancer have mainly been derived from the life span study of Japanese atomic bomb survivors, radiological accidents survivors, and radiotherapy patients follow‐up.^(^ ^44^ ^)^ These data span a dose range of 0.1 to 2.5 Gy. Previous studies have suggested that there is a lot of uncertainty about the risk values for radiation doses outside the above range.^(^ ^44^ ^,^ ^45^ ^)^ The organs or organ parts included in the irradiated area may receive a radiation dose up to 50.4 Gy during treatment of breast cancer, whereas the maximum out‐of‐field organ dose was limited to about 1.6 Gy. Based on the above data, any organ part exposed by the primary beam was not taken into account during MC simulations, in accordance with Bednarz and Xu.^(^ ^33^ ^,^ ^34^ ^)^ The exclusion of the heavily irradiated areas from dose calculations could lead to a dosimetric dataset concerning only the organs exposed by intermediate and low‐dose levels. This dataset might be combined with organ‐specific cancer risk coefficients, to estimate the risk for secondary malignancies after breast radiotherapy.

Recently, the ICRP issued a statement about tissue reactions, particularly those with late manifestation.^(^ ^46^ ^)^ New epidemiological data suggest that the dose threshold for circulatory disease may be 0.5 Gy to the brain and heart. The Commission recommends that optimization should be performed in all exposure situations and for all exposure categories that might take place. This study has demonstrated that mean heart dose due to radiotherapy for breast cancer may exceed the value of 1.6 Gy. This clearly implies that parts of the heart receive even higher radiation dose due to the dose gradient towards the therapeutic field boundaries. Physicians need to keep in mind the high heart exposure and the associated risk, especially when treating young patients.

All MC simulations presented here were carried out using a mathematical phantom. The phantom was modified extensively to include all OARs defined by the ICRP^(^ ^15^ ^)^ and to remove the left arm from the irradiation field. If the left arm remained attached to the phantom's trunk, the RBM and BS in the arm region would receive much higher doses than those observed in real patient's treatment. Organ dose calculations were obtained by using two different dimensions of medial and lateral fields corresponding to two different breast sizes representing a small and a large breast, according to the literature.^(^ ^29^ ^,^ ^30^ ^)^ Further research is required to provide dose data for more field sizes that may be applied in clinical practice. Moreover, the dependence of organ dose upon the wedge introduction into the primary beam needs to be investigated.

The two‐variable (RBM fraction and mass‐energy absorption coefficient weighting) approach to the calculation of RBM dose is limited by the fact that it assumes existent electron equilibrium inside the trabecular spongiosa, which may not be exactly the case.^(^ ^24^ ^)^ More data regarding dose distribution inside the osseous microstructures, as well as the distribution of the BS in the human skeleton, are required to achieve more accurate dose determination to the BS.

In this study, a standard size mathematical phantom was used to assess the doses to peripheral organs due to breast cancer radiotherapy. This phantom was selected because it can be easily modified either to represent missing organs of interest or to change the breast size. The phantom used in the current work represents a taller patient with a lower body mass index compared to the average European woman.^(^ ^47^ ^,^ ^48^ ^)^ The effect of body size on dose to internal organs has not been investigated. Further research is needed to adequately answer the clinically interesting question of the effect of patient body size on organ doses. Organ dose calculations were performed by using a fixed SSD of 100 cm in all simulations. This practice can provide the most conservative dose estimations taking into account that the adoption of a source‐to‐axis distance (SAD) setup may result in lower out‐of‐field doses.^(^ ^49^ ^)^ It also has to be pointed out that, due to the inherent accuracy of MC simulation models, the mathematical phantom could be positioned exactly in the same position with that of an immobilized patient. This represented the ideal immobilization of the patient. However, in an actual radiotherapy setup, some reasonable errors in patient immobilization would introduce uncertainties in organ dose estimations. Internal organ motion and the breathing process in actual radiotherapy would also introduce uncertainties. Estimations of the effect of the breathing process during breast radiotherapy have been documented in the literature.^(^ ^50^ ^,^ ^51^ ^)^ To the best of our knowledge, there are no studies assessing the effect of immobilization error, breathing process, and internal organ motion on dose to organs far away from the edges of the irradiation field. Furthermore, out‐of‐field organ doses need to be assessed for intensity‐modulated radiotherapy of breast cancer, which might be associated with an increase of radiation induced second cancers.^(^ ^44^ ^)^

## V. CONCLUSIONS

In the current study, a cascaded MC model of a medical linac producing 6 MV X‐rays was developed. The accuracy of the generated model was verified against analytical ionization and TLD measurements. The modeled beam was combined with a modified mathematical humanoid phantom to provide accurate and efficient organ dose calculations attributable to radiation therapy for breast cancer. The obtained organ dose data could be employed by radiotherapists, clinicians, and medical physicists whenever questions about the risk for second cancer induction arise in clinical practice. Accurate knowledge of the risk for radiotherapy‐induced secondary malignancies may be of value in the follow‐up studies of young female patients who have undergone breast cancer treatment.
